# Evaluation of microstructural changes in the brain in transfusion dependent thalassemia patients with advanced magnetic resonance imaging techniques

**DOI:** 10.1007/s00234-024-03414-y

**Published:** 2024-07-08

**Authors:** Barış Genç, Kerim Aslan, Memiş Hilmi Atay, Hüseyin Akan

**Affiliations:** 1 Department of Radiology, Ondokuz Mayıs University Faculty of Medicine, Samsun, Turkey; 2 Department of Hematology, Ondokuz Mayıs University Faculty of Medicine, Samsun, Turkey

**Keywords:** Transfusion-dependent thalassemia, Diffusion tensor imaging, Tract-based spatial statistics, Fixel-based analysis

## Abstract

**Purpose:**

Transfusion-dependent thalassemia (TDT) is associated with iron accumulation in the body and an increased tendency for thrombosis. With the increased life expectancy in these patients, the detection of neurocognitive complications has gained importance. This study investigates the microstructural changes in TDT patients using advanced diffusion MRI techniques and their relationship with laboratory parameters.

**Methods:**

The study included 14 TDT patients and 14 control subjects. Tract-based spatial statistics (TBSS) were used to examine differences in DTI parameters such as fractional anisotropy (FA), mean diffusivity (MD), axial diffusivity (AD), and radial diffusivity (RD) in thalassemia patients using multi-shell DWI images. The mean kurtosis (MK) difference was investigated using diffusion kurtosis imaging. Fiber density (FD), fiber cross-section (FC), and fiber density and cross-section (FDC) differences were examined using fixel-based analysis. In the patient group, correlative tractography was used to investigate the relationship between DTI parameters and platelet (PLT) and ferritin levels.

**Results:**

Increase in RD and MD was observed, particularly in the white matter tracts of the corona radiata in patient group. Additionally, an increase in AD was detected in a limited area. Correlative tractography in thalasemia patients showed a positive correlation between increases in RD, MD, and AD with PLT and ferritin. Fixel-based analysis demonstrated a dispersed distribution in white matter fibers, with a more pronounced decrease in FD, FC, and FDC in the internal capsule.

**Conclusion:**

There is widespread involvement in the white matter and fiber tracts in thalassemia patients, which is highly correlated with thrombotic parameters.

## Introduction

Beta-thalassemia are a group of diseases characterized by ineffective erythropoiesis and microcytic anemia caused by abnormal beta-globin chain production. This disease spectrum ranges from mild forms such as thalassemia minor, which is characterized only by mild microcytic anemia, to severe forms such as thalassemia major, which requires regular blood transfusions and can lead to complications such as liver and heart failure, arrhythmias, and even death [1, 2].

In the 1970s, only a third of individuals with thalassemia major could reach the age of 16, with many succumbing to chronic anemia and its complications. Subsequent improvements in lifespan were achieved through transfusions; however, patients then began to die from cardiac complications and arrhythmias due to accumulated iron. Now, with the use of potent chelators, we are able to manage cardiac complications, significantly increasing the life expectancy of individuals with thalassemia [3]. Yet, as life expectancy has increased, the detection of cognitive and neurological problems associated with the disease in older age has gained importance [4]. Accordingly, it is crucial to intervene prophylactically during childhood before the onset of cognitive complications predicted in later life in this congenital disease.

One of the fundamental problems in individuals with thalassemia, especially more pronounced in the intermedia group, is hypercoagulability [5]. These patients often undergo splenectomy at an early age, which leads to increased thrombocytosis. Moreover, the platelets in these patients are more prone to thrombosis as they circulate with hemolyzed erythrocytes in the blood. Elevated ferritin levels in the blood increase the risk of thrombosis in these patients, and iron chelation can reduce this risk [6]. Studies have shown that patients with transfusion-dependent thalassemia (TDT) exhibit increased white matter lesions on T2-FLAIR images, a finding that is prominent in thalassemia intermedia (TI) patients and associated with splenectomy [7]. Research in sickle cell anemia patients has revealed microstructural changes through diffusion tensor imaging (DTI) even when conventional MRI appears normal [8]. Thus, in addition to conventional MRI sequences, diffusion-weighted imaging could provide additional insights into white matter damage in these patients.

Determining the presence or absence of this risk of white matter lesions is important because preventing these lesions, along with the increased life expectancy, could help avoid the increased risk of dementia in these patients [9]. If such a risk exists, treatment options to address the hypercoagulation in small vessels from childhood could become relevant [10]. The first study by Russo and colleagues in Italy using DTI and tract based spatial statistics (TBSS) found no microstructural changes in the white matter of these patients [11]. However, this study did not perform fixel-based analysis nor investigated the relationship between laboratory parameters and DTI parameters. Fixel-based analysis can reveal microstructural changes not visible through TBSS [12]. Additionally, the relationship between thrombotic risk factors and diffusion parameters is crucial for preventing these risk factors.

Our study aims to assess microstructural changes in patients with transfusion-dependent thalassemia using a multimodal diffusion-weighted imaging approach. This involves utilizing DTI parameters along with multi-shell diffusion kurtosis imaging and fixel-based analysis. Additionally, we aim to understand the relationship between thrombotic risk factors and microstructural changes in the brain.

## Materials and methods

The design of the study was observational. Ethical approval for the study was obtained (Ondokuz Mayıs University Clinical Research and Ethics Committee: 2021/422). Before each examination, all subjects were fully informed and provided written informed consent. The STROBE (Strengthening the Reporting of Observational Studies in Epidemiology) guidelines were adhered [13].

### Participants and clinical data

The study included fifteen transfusion-dependent thalassemia patients aged 18–65 years, without a history of stroke or neurological disease, receiving regular blood transfusions at our hospital; fourteen of these patients were able to tolerate the MRI procedure. After the MRI review, it is planned to exclude patients with evidence of a previous stroke in conventional MRI sequences (T1, T2, FLAIR) from the study. As a control group, fourteen healthy volunteers, matched for age and gender, who had undergone a complete blood count (CBC) for any reason within the last year, were included. A CBC was performed for the patients before blood transfusion, and MRI scans were conducted on the same day.

### MRI imaging

The MRI examinations were performed using a 3 T scanner (Ingenia, Philips Healthcare, Best, The Netherlands) with a 32-channel head coil. All examinations included a 3D T1-weighted conventional gradient echo (3D T1-fast field echo) sequence without gadolinium (160 contiguous sagittal slices with an in-plane voxel resolution: 1 × 1 × 1 mm; repetition time [TR]/echo time [TE], 7.9/3.5 ms [ms]; section thickness, 1 mm; number of excitations [NEX], 1), 3D fluid attenuation inversion recovery (FLAIR) coronal (TR/TE/Inversion time, 4800/361/1650 ms; section thickness, 1 mm; NEX, 1); 3D T2-weighted (T2W) axial turbo spin-echo sequence (TR/TE, 2500/261 ms; section thickness, 0,6 mm; NEX, 1), axial susceptibility weighted imaging (SWI) (TR/TE, 31/0 ms; section thickness, 0,3 mm; NEX, 1). Diffusion-weighted images were obtained with spin-echo and echo-planar sequences with 3 b values (0, 1000, 2500) and 64 directions for each b value different from 0. Other diffusion parameters were TR/TE, 4636/117 ms; section thickness, 2.3 mm; FOV, 240 × 240 mm^2^, matrix, 128 × 128; NEX, 1.

### Diffusion-weighted i̇maging analysis

#### Tract based spatial statistics

Initially, "dwidenoise" command within mrtrix 3.0.2 was used for denoising [14], followed by distortion correction using FMRIB Software Library (FSL) TOPUP [15]. Subsequently, eddy current artifacts were removed with FSL eddy [16, 17]. DTI images (fractional anisotrophy [FA], mean difusivity [MD], radial difusivity [RD], axial difusivity [AD]) were obtained using images at b = 0 and b = 1000 s/mm^2^, while DKI maps (MK) utilized multi-shell b values (b = 0 and b = 1000 s/mm^2^, b = 2500 s/mm^2^). DKI and DTI maps were acquired using dti_fit in FSL. Similar to previous articles, mean FA skeleton images for TBSS were created and voxel-wise statistics were performed with randomise [18]. Age and gender were used as covariates. Statistically significant voxels (*p* < 0.05) were presented using the "autoaq" command in FSL with the Johns Hopkins University (JHU) Tractography atlas.

### Fixel-based analysis

For the analysis of fibre density and cross-section in diffusion-weighted images, the standard multi-tissue constrained spherical deconvolution (CSD) using fixel-based analysis pipeline within Mrtrix 3.0.2 was utilized [19]. This pipeline is detailed on the Mrtrix website: https://mrtrix.readthedocs.io/en/latest/fixel_based_analysis/mt_fibre_density_cross-section.html. Uncorrected *p* < 0.005 was considered statistically significant.

#### Correlational tractography

To investigate the relationship between hemoglobin, platelet count, and ferritin levels with the significant parameters obtained from TBSS analysis of DTI parameters, correlational tractography and diffusion MRI connectometry analysis were conducted using DSI Studio.

Non-parametric Spearman correlation analysis was employed with age as a covariate. A T score threshold of 2.5 was used for selecting local connectomes, with a tract length threshold of 20 mm [20]. To prevent false connectomes, the Topology-Informed Pruning (TIP) algorithm was used [21]. The false discovery rate (FDR) was estimated from a total of 2000 randomized permutations applied to the group label to obtain the null distribution of the track length. FDR < 0.05 was considered statistically significant.

#### Statistical analysis

Demographic and clinical information and the intensity values of subcortical grey matter structures in magnitude images were compared using the Shapiro–Wilk test to assess normal distribution, followed by the T-Test or Mann–Whitney U test.

## Results

### Clinical and demographic information

The study included twelve patients with thalassemia intermedia and two with thalassemia major, thirteen of whom were splenectomized. The patients were 34.73 ± 9.42 years old, while the control group was 38.1 ± 15.0 years old, with no significant difference between the groups (*p =* 0.834). Both the patient and control groups consisted of twelve males and two females. The hemoglobin value in the control group was 13.0 ± 1.0 g/dl; in contrast, the patient group had a hemoglobin value of 7.97 ± 1.1 g/dl, which was statistically significantly lower (*p* < 0.001). Platelet count in the patient group was 559 ± 220 × 10^3^/µl; in the control group, it was 270 ± 70 × 10^3^/µl, indicating an increase in the patient group (*p* < 0.001). Ferritin levels were 195 ± 70 ng/ml in the control group and 1069 ± 843 ng/ml in the patient group, showing an increase in the patient group (*p* < 0.001) (Table 1). In conventional MRI sequences, no findings suggestive of stroke were detected in the thalassemia patient group or in healthy controls.

**Table 1:** Demographic characteristics and laboratory results

	Healthy Control (*n* = 14)	Thalassemia (*n* = 14)	*p* value
Women (No.) (%)	2 (14)	2 (14)	1
Age (yr)	38.1 ± 15.0	34.7 ± 9.4	0.83
Hemoglobin (g/dl)	13.0 ± 1.0	8.0 ± 1.1	< 0.001
Platelet count (× 10^3^/µl)	270 ± 70	559 ± 220	< 0.001
Ferritin (ng/ml)	195 ± 70	1069 ± 843	< 0.001

### TBSS

**Fig. 1:**
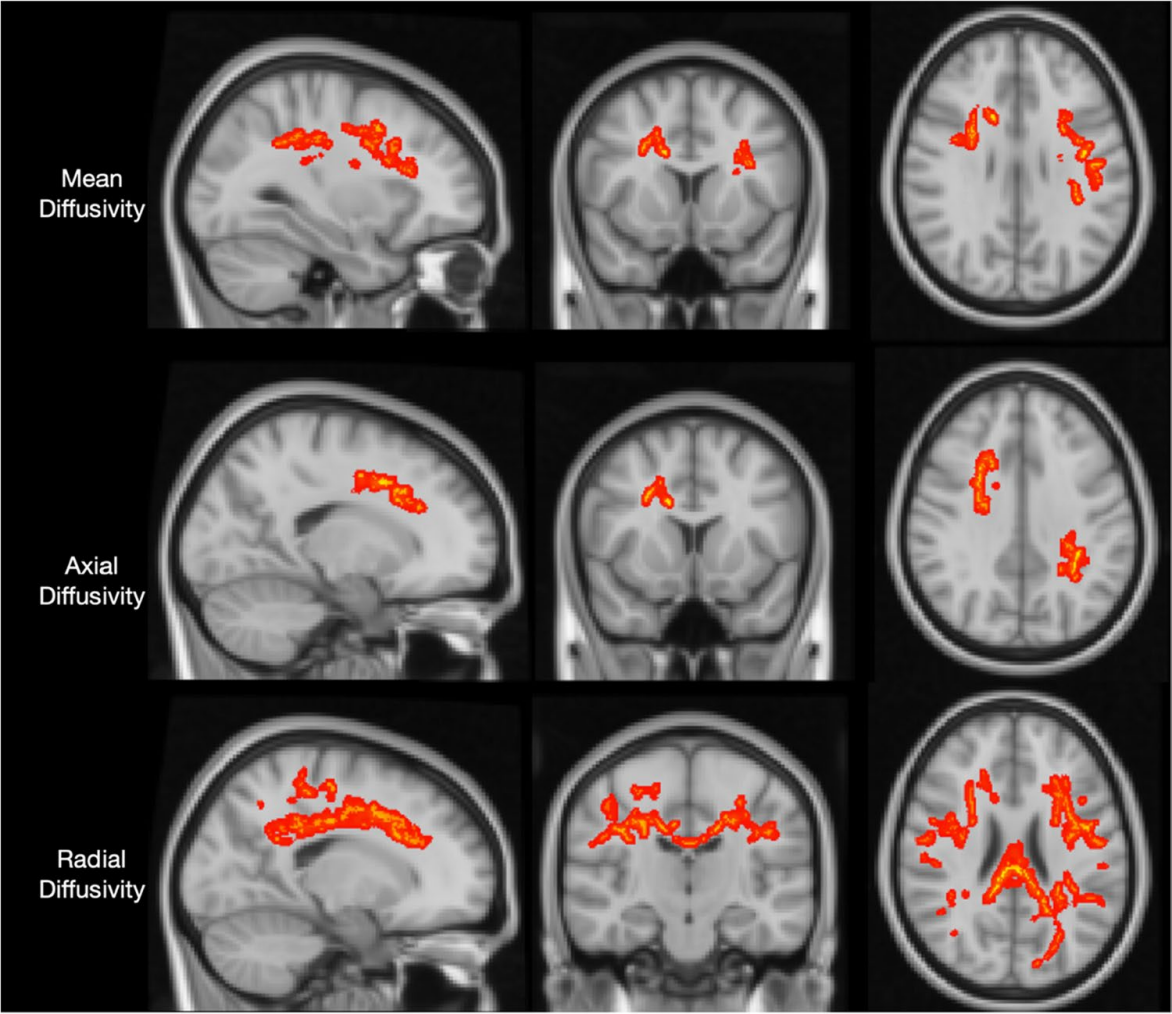
Tract-Based Spatial Statistics (TBSS) results show areas with increased axial diffusivity, mean diffusivity, and radial diffusivity in Thalassemia patients (*p* < 0.05 FWE)

Thalassemia patients showed a significant increase in radial diffusivity in the genu-splenium and body sections of the corpus callosum, the anterior limbs of the bilateral internal capsule, and the bilateral thalamic radiation fibers including optic radiation fibers, and in the bilateral superior longitudinal fasciculus (SLF) (*p* < 0.05 Family-wise error [FWE]). Thalassemia patients exhibited an increase in MD in the body of the corpus callosum and bilateral SLF fibers (*p* < 0.05 FWE). There was an increase in AD in the corpus callosum body and splenium, bilateral SLF, and optic radiation fibers in thalassemia patients (*p* < 0.05 FWE). No difference was observed in FA and MK between the groups (*p* > 0.05 FWE) (Fig. 1) (Table 2).

**Table 2:** Tract-Based Spatial Statistics (TBSS) results thalassemia patients vs. healthy controls

Cluster Index	Voxels	*p* value	Peak MNI Coordinates, mm (x, y, z)	Tracts
Radial diffusivity				
1	1496	0.004	-29, -49, 34	Left corona radiata, left posterior thalamic radiation, left SLF
2	1316	0.006	26, 17, 16	Right anterior and superior corona radiata, right SLF, body of corpus callosum
3	829	0.006	-47, 3, 14	Left SLF
4	320	0.006	-29, 26, 16	Left SFOF
5	257	0.006	34, -16, 28	Right SLF
6	232	0.006	44, -5, 25	Left SLF
7	232	0.006	36, -49, 29	Right SLF
Mean diffusivity				
1	780	0.04	-34, -4, 22	Left superior corona radiata, left SLF
2	567	0.04	27, 3, 30	Body of corpus callosum, superior corona radiata
3	259	0.04	-27, -45, 37	Left SLF
4	211	0.044	-47, -13, 25	Body of corpus callosum, right anterior and superier corona radiata
5	136	0.046	15, 13, 28	Left SLF
Axial diffusivity				
1	665	0.032	-28, -45, 34	Left posterior corona radiata, left SLF
2	613	0.032	26, 8, 32	Right anterior and superior corona radiata, body of corpus callosum

*MNI* Montreal Neurological Institute, *SLF* Superior longitudinal fasiculus, *SFOF* Superior fronto-occipital fasciculus

### Fixel-based analysis

**Fig. 2:**
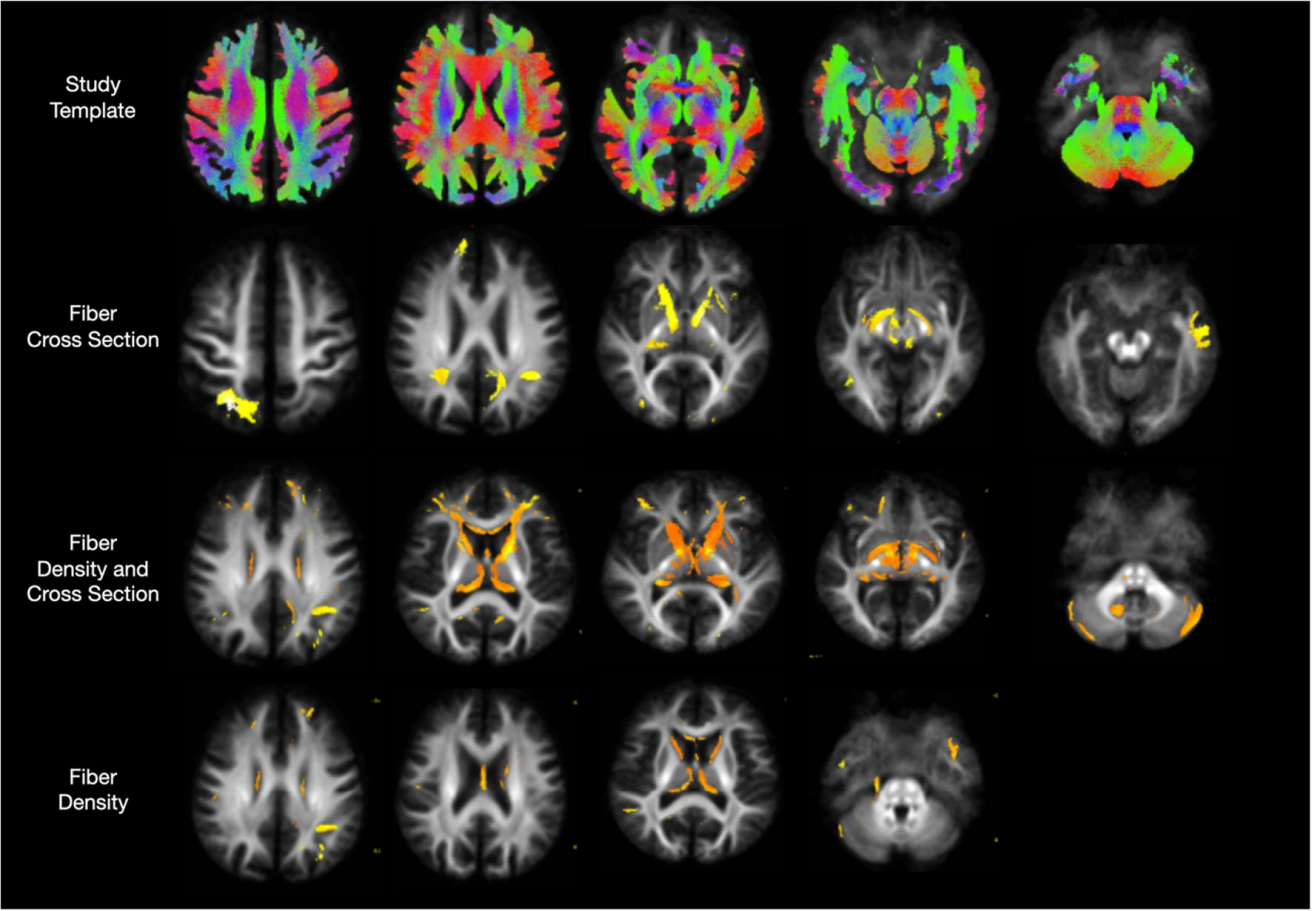
Areas of significant redulction in fiber density and cross-section, fiber density, fiber bundle cross-section (FC) in patients with Thalassemia compared to healthy control. Only significant fixels were shown (*p* < 0.005 uncorrected)

In the fiber cross-section analysis, a decrease was detected in the right superior parietal, angular gyrus, right cingulum, bilateral anterior limb of the internal capsule, and left temporal. A decrease in fiber density was observed in the bilateral inferior temporal, left superior parietal in the corpus callosum, and superior cerebellar peduncle. A decrease in FDC was detected in the cerebral parenchyma white matter, internal capsule, genu and splenium of the corpus callosum, and bilateral superior and middle frontal white matter areas (*p* < 0.005) (Fig. 2).

### Correlational tractography

**Fig. 3:**
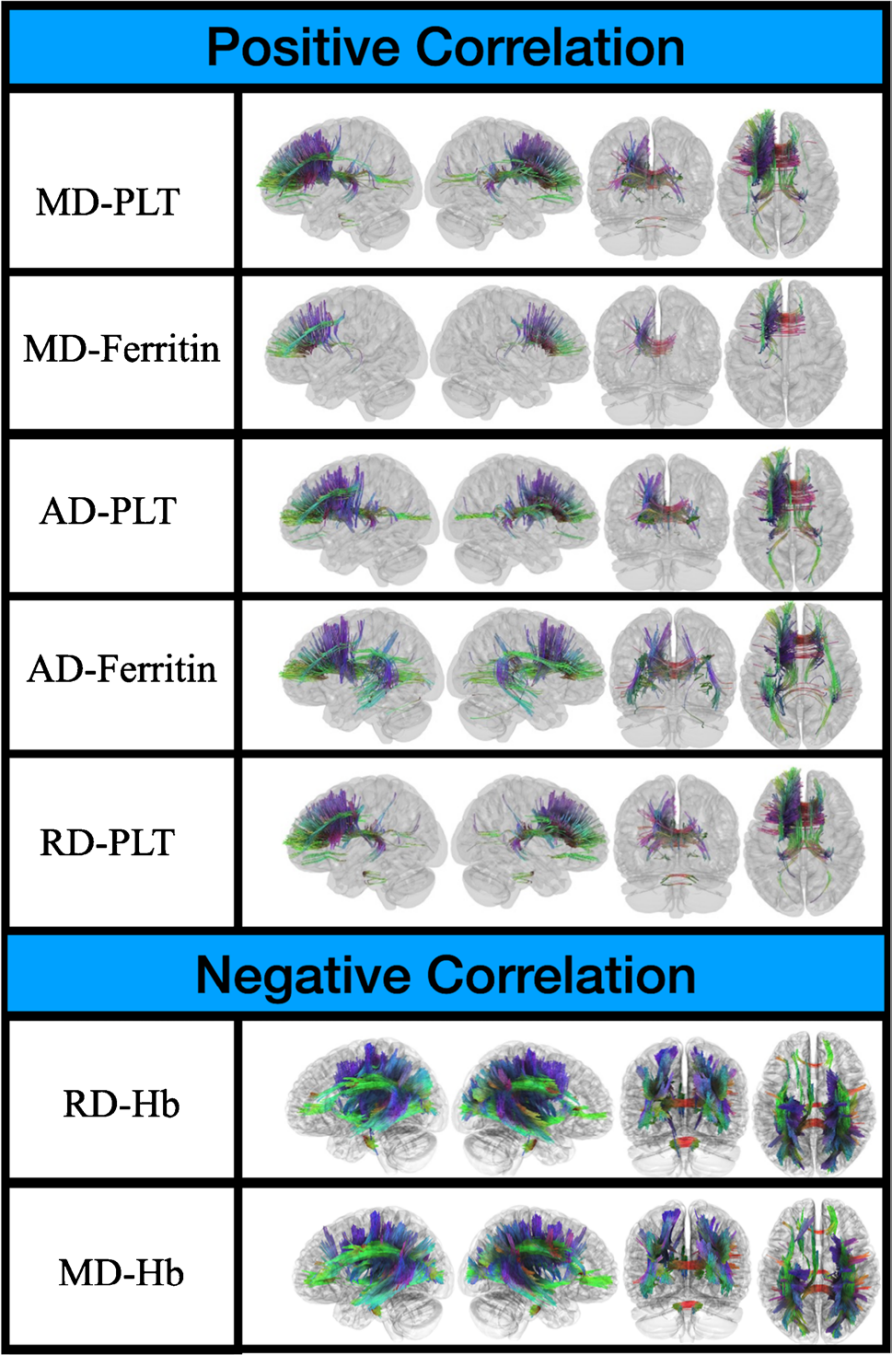
Correlational tractography results of thalassemia patients

Positive correlations were found between MD and AD values with PLT and ferritin values in the forceps minor, corpus callosum body, left superior and anterior thalamic radiation, left dentatorubrothalamic tract, left corticostriatal tract, left SLF2, left cingulum, right fornix, left corticopontine tract, right superior thalamic radiation fibers. Positive correlations were also observed between radial diffusivity and PLT in the forceps minor, corpus callosum body, left anterior and superior thalamic radiation fibers, right fornix, left dentatorubrothalamic tract, left fornix, left cingulum, left corticostriatal tract, right superior thalamic radiation fibers (FDR < 0.05). No correlation was found between RD and ferritin (FDR > 0.05) (Fig. 3). The MD and RD values in the supratentorial white matter tracts have shown a widespread negative correlation with the hemoglobin count. These are particularly evident in the posteriorly located splenium of the corpus callosum, optic radiation fibers, superior longitudinal fasciculus, corticospinal tract, and thalamic radiation fibers (FDR < 0.05). No correlation was found between AD and hemoglobin (FDR > 0.05) (Fig. 3).

## Discussion

In this study, microstructural changes in patients with thalassemia were investigated using advanced diffusion MRI techniques. Our findings reveal widespread increases in radial diffusivity in the white matter of thalassemia patients, along with more limited increases in axial and mean diffusivity, and for the first time, demonstrate a widespread decrease in fiber cross section along with FDC, FC and FD in white matter fibers. Our correlational tractography results have, for the first time, shown that the number of platelets (PLT) and the amount of ferritin in the blood are highly related to damage in fiber tracts in these patients.

A study on asymptomatic individuals with sickle cell anemia found an increase in T2/FLAIR hyperintense asymptomatic white matter lesions consistent with increased silent cerebral ischemia in patients with sickle cell anemia compared to normals. Even when conventional MRI images were normal, TBSS analysis found increases in RD and MD consistent with white matter damage in sickle cell anemia patients not detected by conventional MRI [8]. Knowing whether patients with TDT also have a risk of silent cerebral infarct is important for deciding whether to administer antithrombotic drugs [10]. Conventional MRI studies in patients with TDT have found an increase in white matter lesions, and this risk has been calculated to be higher in splenectomized TI patients [5, 7, 22, 23]. Our study has shown increases in MD, RD, and AD in TDT patients, supporting white matter damage in these patients in a similar manner to previous studies.

Studies have shown that in transfusion-dependent thalassemia patients, a ferritin level above 1000 ng/dl increases the risk of thrombotic events by 1.86 times [24]. A PLT count above 500,000/µl is also one of the most significant risk factors for thrombosis [24]. However, the changes they cause in the brain have not been quantitatively correlated. Our study is the first to show a positive correlation between the PLT count and MD, RD, AD values in white matter fibers. Additionally, ferritin showed and a positive correlation with MD in more limited areas, and a more widespread positive correlation with AD. All these findings indicate that the increase in ferritin and PLT is related to damage in the white matter of thalassemia patients.

Changes in TBSS with DTI parameters in TDT patients have, to our knowledge, only been investigated by Russo and colleagues, but no significant differences in DTI parameters were found [11]. Although these findings completely contradict ours, it is unknown how many of the TDT patients in that study had thalassemia major (TM) versus TI. 86% of the TDT patients in our study have TI. It is known that TI patients carry a 4.5 times higher risk of thrombotic events than TM patients. Furthermore, while 54.5% of the transfusion-dependent thalassemia patients in Russo and colleagues' study were splenectomized, 92.8% in our study were splenectomized. Being splenectomized increases the risk of thrombosis by 6.59 times in thalassemia patients. The difference in the rate of splenectomized patients and the proportion of TI patients may explain the differences in study findings. Future multicenter studies that separately evaluate TI and TM patients could clarify these differences. In a recent study by Zacarías et al., which included patients with thalassemia, a significant decrease in FA, particularly in the frontoparietal regions, was demonstrated in a group of patients with chronic anemia. This reduction in FA was also associated with decreased structural connectivity [25]. Another study revealed that anemia alone is associated with cognitive impairment [26]. This indicates that white matter damage in thalassemia patients is not solely related to thrombotic events, but that existing anemia itself also induces white matter damage. Indeed, our study found a widespread negative correlation between RD, MD and hemoglobin in the white matter tracts of thalassemia patient. All these findings suggest that there are multiple risk factors that could affect the white matter in patients with thalassemia.

In the animal study conducted by Song and colleagues, an increase in RD began during the days when myelin loss was initiated in ischemic optic nerves, and a relationship was found between myelin damage and RD [27]. Microvascular damage is associated with myelin loss, and the widespread increase in RD observed in patients with thalassemia may be related to RD damage linked to thrombosis. Indeed, in correlational tractography, RD is highly related to PLT, another indicator of thrombosis. AD is affected both by axons and myelin [27]. While axonal damage reduces AD, myelin damage leads to an increase in AD. In fact, fixel-based analysis detected a reduction in density and diameter in areas that showed an increase in RD but not in AD, which might explain why some fibers, such as the corona radiata and corpus callosum, exhibit increased RD without a corresponding increase in AD. Moreover, the absence of changes in FC, FD and FDC in bilateral superior longitudinal fasciculus (SLF), corpus callosum areas where both AD and RD increase could support the presence of pure myelin damage and the secondary increase in AD.

TBSS is inadequate in analyzing the cumulative effect of multiple different fiber populations within voxels. Fixel-based analysis has been developed in recent years to overcome this, and studies have shown that microstructural changes not detected by DTI or TBSS can be revealed by fixel-based analysis. While TBSS shows disease-related changes in white matter areas, especially in the centrum semiovale plane, reductions in FDC and FC in white matter fibers in the corona radiata plane and cerebellum can be shown with fixel-based analysis. A PET-CT study in thalassemia patients has shown decreased FDG uptake in left temporal and parietal white matter areas not seen in conventional MRI, aligning with our findings of decreased FC in bilateral parietal and left temporal white matter [28]. DKI is an imaging technique that measures the non-Gaussian distribution of water diffusion in tissues, and is associated with tissue heterogeneity. Complex structures such as axons, dendrites, and myelin can increase the mean kurtosis (MK) value [29, 30]. In the context of stroke, an increase in MK is also observed [30]. In patients with thalassemia, myelin and axonal damage secondary to thrombosis can lead to a decrease in MK, while chronic microthrombotic cellular damage might contribute to an increase in MK. This complex situation might explain why, in thalassemia patients, there is an increase in mean diffusivity (MD) without a corresponding increase in MK. Such dynamics underscore the intricate relationship between tissue architecture disruption and diffusion imaging metrics, revealing the nuanced impact of pathological processes on imaging signatures.

With the increase in the expected lifespan of thalassemia patients, preventing silent cerebral ischemias that can cause cognitive decline in later life has become important. A study in Iran showed that regular use of acetylsalicylic acid slowed the formation of white matter damage [10]. It is also recommended that patients at risk be followed up every three years with conventional MRI sequences [31, 32]. Our findings also suggest widespread white matter damage in these patients, likely related to PLT and possibly thrombosis. Therefore, using antithrombotic drugs in patients at risk and monitoring these patients with diffusion-weighted imaging in addition to conventional MRI sequences may prevent future cognitive decline.

Our study has various limitations. It is a single-center study, and the longitudinal course of this white matter damage was not evaluated. Since cognitive functions were not assessed, the findings could not be related to cognitive impairment. Thalassemia affects multiple systems and involves multi-organ involvement. The changes in the brain could be secondary to the systemic organ involvement of the disease, but evaluating this requires extensive longitudinal studies. Our patient cohort is not sufficiently large to generalize the results, necessitating further studies with a broader scope to validate these findings. Although our patient group consists largely of thalassemia intermedia patients, it is heterogeneous. The courses of thalassemia intermedia and major are different, necessitating studies that evaluate these diseases separately.

In conclusion, there is widespread white matter damage in transfusion-dependent thalassemia patients. This damage has caused disruptions in diffusion tensor parameters as well as in the density of white matter fibers and fiber cross section. The alterations in diffusion tensor parameters are related to increases in platelet and ferritin levels. Our results indicate that these patients should be carefully monitored for silent brain damage, and preventive treatments should be considered.
